# Risk Factors Associated With Major Adverse Cardiovascular Events and Malignancies in Patients With Rheumatoid Arthritis in a Real‐World Setting in Japan

**DOI:** 10.1111/1756-185X.15448

**Published:** 2024-12-09

**Authors:** Kunihiro Yamaoka, Naonobu Sugiyama, Masato Hoshi, Joo‐Young Jo, Kichul Shin, Toshitaka Hirano

**Affiliations:** ^1^ Department of Rheumatology and Infectious Diseases Kitasato University School of Medicine Sagamihara Japan; ^2^ Pfizer Japan Inc Tokyo Japan; ^3^ Pfizer Pharmaceuticals Korea Ltd Seoul South Korea; ^4^ Division of Rheumatology Seoul Metropolitan Government‐Seoul National University, Boramae Medical Center Seoul South Korea

**Keywords:** MACE, malignancy, real‐world data, rheumatoid arthritis, risk

## Abstract

**Aim:**

To identify risk factors associated with major adverse cardiovascular events (MACE) and malignancies in patients with rheumatoid arthritis (RA) using real‐world data from Japan.

**Methods:**

This cohort study used the Real World Data database of medical institutions in Japan. Eligible patients (January 2013–December 2021) had ≥ 1 RA diagnosis, were aged ≥ 18 years, prescribed ≥ 1 antirheumatic drug, had no psoriasis diagnosis, and had a record postindex. Patients had no myocardial infarction/stroke ≤ 31 days (MACE cohort) or malignancy < 1 year (malignancy cohort) before index. Cohorts were determined by incidence of initial MACE or malignancy. Known/exploratory variables were selected using Cox regression models.

**Results:**

Across MACE (*n* = 16 012) and malignancy (*n* = 14 545) cohorts, most patients were female and aged ≥ 65 years. Overall, 214 MACE per 43964.7 patient‐years (incidence rate 0.49 per 100 patient‐years) and 315 malignancies per 40251.6 patient‐years (incidence rate 0.78 per 100 patient‐years) occurred. Male sex, older age (≥ 65 years), hypertension, renal disease, cerebrovascular disease, and prior X‐ray examination were significantly associated with increased MACE risk. Male sex, older age (≥ 50 years), nonsteroidal anti‐inflammatory drug use, emphysema, serious infection, malignancy history, and prior X‐ray examination were significantly associated with increased malignancy risk. Conversely, glucocorticoid use and fracture diagnosis were significantly associated with reduced malignancy risk.

**Conclusion:**

In patients with RA in Japan, male sex, older age, and prior X‐ray examination were associated with increased MACE and malignancy risk.

## 1 Introduction

Rheumatoid arthritis (RA) is a chronic inflammatory disease that primarily affects the joints [1] and has an estimated prevalence of 0.6%–1.0% in Japan [2, 3, 4]. Patients with RA also experience extra‐articular manifestations and are at increased risk of developing comorbidities [1], such as cardiovascular disease [5], and certain malignancies than the general population [6, 7]. In studies of Western populations with RA, cardiovascular disease has been highlighted as one of the leading causes of mortality [8, 9], while in a cohort of Japanese patients with RA, malignancies were identified as one of the most common causes of mortality [10].

Several traditional risk factors for cardiovascular disease, such as smoking, hypertension, diabetes, and higher body mass index, have also been identified as risk factors for the development of cardiovascular disease in patients with RA [11, 12, 13], and RA‐associated systemic inflammation (based on disease activity) is thought to be an additional independent risk factor [13, 14]. Inflammatory mechanisms associated with RA have been postulated as risk factors for the increased risk of lymphoma [15], while older age and the use of methotrexate and tacrolimus have been identified as risk factors for lymphoma in patients with RA in Japan [6]. Furthermore, in a study of Australian patients with RA or psoriatic arthritis, the use of methotrexate was associated with an increased risk of nonmelanoma skin cancer (NMSC) [16], while a systematic review and meta‐analysis of patients with RA suggested that use of tumor necrosis factor inhibitors (TNFi) was also associated with an increased risk of NMSC [17].

Most studies assessing the risk of cardiovascular disease and malignancies in patients with RA have, to date, primarily included global and/or Western populations; however, incidence of these safety outcomes could vary across different geographic locations. For example, in a postauthorization safety study, where it was shown that there was an increased rate of major adverse cardiovascular events (MACE) and malignancies in cardiovascular risk‐enriched patients with RA receiving tofacitinib (an oral Janus kinase [JAK] inhibitor) versus TNFi, the risk of MACE/malignancies with tofacitinib was shown to be higher among those in North America versus the rest of the world [18]. This may be due to the increased prevalence of differential risk factors, such as age ≥ 65 years and ever smoking, among patients in North America [18, 19]. However, an increased risk of MACE and malignancies in patients with RA treated with JAK inhibitors versus TNFi, or with tofacitinib versus TNFi, were not observed in real‐world studies of US claims data and international databases [20, 21]. In addition, in relation to the corresponding proportion of the global population, the incidence of cancer is thought to be higher in Europe and North America than in Africa and Asia [22]. Therefore, an improved understanding of the incidence of MACE and malignancies and underlying risk factors for these outcomes, in real‐world populations of patients with RA, across specific geographic locations is required.

In this observational cohort study, we sought to identify risk factors for MACE and malignancies (including NMSC) in patients with RA using the Real World Data database of medical institutions in Japan.

## 2 Materials and Methods

### 2.1 Study Design and Data Source

This was a cohort study of patients with RA using a real‐world database of medical institutions in Japan. Two cohorts of patients with RA were created: (1) MACE cohort and (2) malignancy cohort.

Patients with RA were identified using the Real World Data database maintained by the Health, Clinic, and Education Information Evaluation Institute (Kyoto, Japan) with support from Real World Data Co. Ltd. (Kyoto, Japan). The Real World Data database is a nationwide administrative database of 25 million patients from 229 medical institutions in Japan (as of July 2022). The database includes information from electronic medical records, claims data, and discharge abstract data relating to patient demographics, diagnoses, prescriptions, procedures, and laboratory test results.

### 2.2 Patients

Eligible patients (January 2013–December 2021) had ≥ 1 RA diagnosis, were aged ≥ 18 years (on index date), prescribed ≥ 1 antirheumatic drug (disease‐modifying antirheumatic drugs [DMARDs] or glucocorticoids) and had no psoriasis diagnosis [23] within ≤ 28 days of RA diagnosis, and had ≥ 1 record after the index date. Hospitals were limited to diagnostic procedure combination hospitals.

In the MACE cohort, patients had no myocardial infarction (MI) or stroke diagnosis in inpatient settings or in outpatient visits ≤ 31 days before index. In the malignancy cohort, patients had no malignancy diagnosis < 1 year before index.

As this study involved anonymized structured data, which according to applicable legal requirements did not contain data subject to privacy laws, obtaining informed consent from patients was not required. According to the ethical guidelines for epidemiologic studies in Japan, informed consent was not required for studies using unlinkable anonymized data [24]. In addition, approvals from an Institutional Review Board/Independent Ethics Committee were not required.

### 2.3 Outcomes

The primary outcomes were the incidence of initial MACE (death from cardiovascular causes, nonfatal MI, or nonfatal stroke) and malignancy during the follow‐up period (up to July 2022). The date of initial MACE was defined as the earliest date among the following criteria: death date during hospitalization due to cardiovascular diseases; nonfatal MI; and nonfatal stroke. Nonfatal MI and stroke were each defined as being the most resource‐consuming diagnosis. The criteria for defining an initial malignancy event were based on those described in a prior study [25]. Each patient was followed from the next day after the index date until the earliest occurrence of an initial MACE or malignancy event or last visit.

### 2.4 Statistical Analysis

Patient demographics and baseline disease characteristics were analyzed descriptively for the MACE and malignancy cohorts. For the respective cohorts, the total number of MACE and malignancy events, total length of follow‐up, and incidence rates (IRs) per 100 patient‐years (PY) were calculated.

For the MACE and malignancy cohorts, a set of known and exploratory risk factors/variables, including demographics, comorbidities, medications, imaging examinations (e.g., X‐rays; occurring 1 year prior to the index date), and laboratory tests were selected using Cox regression models. A sequential variable selection technique with univariate analysis, variance inflation factors, and correlation coefficients was used to determine exploratory risk factors. Crude and adjusted hazard ratios (HRs) and their 95% confidence intervals (CIs) for all independent variables entered in the final model were calculated. Crude HRs were from separate univariate models and adjusted HRs were from the final models after the systematic variable selection procedure. Due to the exploratory nature of the analysis, no adjustment was made for multiplicity. Statistical significance was defined as *p* < 0.05.

Due to missing data for laboratory tests, the primary analysis of the study did not include laboratory tests as risk factors. The following sensitivity analyses were also conducted using the same statistical analyses as above but with the individual sensitivity analysis sets: (1) with laboratory tests included as risk factors; (2) missing‐indicator method (missing data on laboratory test results were identified by the missing‐indicator method where the “missing” category was used); (3) MACE with a wider definition (nonfatal MI and stroke definitions were expanded from being the most resource‐consuming diagnosis, to also including main diagnosis, or admission‐precipitating diagnosis); and (4) incident RA (where the eligibility criteria, “let the latest date satisfied by both RA diagnosis and RA drug prescription after 366 days from the first record date in the database be the index date,” was changed to, “let the latest date satisfied by both RA diagnosis and RA drug prescription be the index date” [for patients who had at least 1 record prior to 12 months before the index date and had at least 1 record within 12 months before the index date]).

## 3 Results

### 3.1 Patients

Of 144 949 patients with an RA diagnosis, 16 012 and 14 545 eligible patients were included in the MACE and malignancy cohorts, respectively (Table 1, Figure S1). Demographics and patient characteristics were generally similar between the MACE and malignancy cohorts. Across cohorts, most patients were female (64.69%–66.90%) and aged ≥ 65 years (68.88%–69.78%), while the most common comorbidities were as follows: hypertension; hyperlipidemia; malignancies (MACE cohort only); and cardiovascular diseases (all ≥ 27.0%; Table 1).

**TABLE 1:** Demographics and patient characteristics.

	MACE cohort (*N* = 16 012)		Malignancy cohort (*N* = 14 545)	
	*n*	%	*n*	%
Female	10 358	64.69	9730	66.90
Age, years				
18–49	1649	10.30	1582	10.88
50–64	3190	19.92	2945	20.25
65–74	4593	28.68	4009	27.56
≥ 75	6580	41.09	6009	41.31
BMI, kg/m^2^				
< 18.5	771	4.82	655	4.50
≤ 18.5–< 25	2771	17.31	2352	16.17
≥ 25	959	5.99	814	5.60
N/A	11 511	71.89	10 724	73.73
Smoking status				
Non‐smoker	3181	19.87	2843	19.55
Smoker	1242	7.76	927	6.37
N/A	11 589	72.38	10 775	74.08
Comorbidities				
Diabetes	2714	16.95	2378	16.35
Cerebrovascular disease	2492	15.56	2632	18.10
Cardiovascular disease	4331	27.05	4271	29.36
Fracture diagnosis	2436	15.21	2250	15.47
Hypertension	6383	39.86	5741	39.47
Hyperlipidemia	4740	29.60	4305	29.60
Malignancy	4762	29.74	—	—
Renal disease	1388	8.67	1181	8.12
Unspecified chronic bronchitis	1212	7.57	1013	6.96
Emphysema	491	3.07	340	2.34
Other COPD	540	3.37	408	2.81
Serious infection	4015	25.07	3496	24.04
Medications				
NSAIDs	9125	56.99	8230	56.58
Glucocorticoids, daily dose, mg				
0	6018	37.58	5644	38.80
> 0–< 5	8112	50.66	7316	50.30
≥ 5–< 10	1126	7.03	947	6.51
≥ 10	756	4.72	638	4.39
bDMARDs	900	5.62	867	5.96
csDMARDs	8505	53.12	7941	54.60
Methotrexate, weekly dose, mg				
0	11 733	73.28	10 472	72.00
> 0–≤ 8	3730	23.30	3554	24.43
> 8	549	3.43	519	3.57
JAK inhibitor	49	0.31	48	0.33
Calcineurin inhibitor	1086	6.78	1002	6.89
Past history				
Deep vein thrombosis	260	1.62	218	1.50
MACE	67	0.42	82	0.56
Malignancy	4731	29.55	3362	23.11
Pulmonary embolism	34	0.21	25	0.17
Prior X‐ray examination	11 100	69.32	9855	67.76

Abbreviations: bDMARD, biologic disease‐modifying antirheumatic drug; BMI, body mass index; COPD, chronic obstructive pulmonary disease; csDMARD, conventional synthetic disease‐modifying antirheumatic drug; JAK, Janus kinase; MACE, major adverse cardiovascular events; *N*, total number of eligible patients; *n*, number of patients with characteristic; N/A, not available; NSAID, nonsteroidal anti‐inflammatory drug.

In both cohorts, the most frequently used concomitant medications were glucocorticoids (61.20%–62.42%), nonsteroidal anti‐inflammatory drugs (NSAIDs; 56.58%–56.99%), and conventional synthetic DMARDs (53.12%–54.6%). A history of MACE was recorded in 0.42% of patients in the MACE cohort, while 23.11% of patients in the malignancy cohort had a history of malignancy. The proportions of patients with missing data regarding body mass index and smoking status were over 70% in both cohorts (Table 1).

### 3.2 MACE and Malignancy Outcomes

In both cohorts, there was an average follow‐up length of 2.8 years. In the MACE cohort, there were 214 MACE recorded over 43964.70 PY (IR 0.49 per 100 PY). Of these, 150 were strokes (44011.24 PY [IR 0.34 per 100 PY]), 35 were MIs (44134.26 PY [IR 0.08 per 100 PY]), 27 were heart failures (44180.63 PY [IR 0.06 per 100 PY]), and 2 were other ischemic heart diseases (44180.71 PY [IR < 0.01 per 100 PY]; Table 2).

**TABLE 2:** MACE and malignancy outcomes.

	MACE cohort (*N* = 16 012)	Malignancy cohort (*N* = 14 545)
Total number of outcome events, *n*	214	315
Total length of follow‐up, years	43 964.70	40 251.60
IR per 100 PY	0.49	0.78
Stroke events^a^, *n*	150	—
Total length of follow‐up, years	44 011.24	—
IR per 100 PY	0.34	—
MI events^a^, *n*	35	—
Total length of follow‐up, years	44 134.26	—
IR per 100 PY	0.08	—
Heart failure events^a^, *n*	27	—
Total length of follow‐up, years	44 180.63	—
IR per 100 PY	0.06	—
Other ischemic heart disease events^a^, *n*	2	—
Total length of follow‐up, years	44 180.71	—
IR per 100 PY	< 0.01	—

Abbreviations: IR, incidence rate; MACE, major adverse cardiovascular events; MI, myocardial infarction; *N*, total number of eligible patients; *n*, number of events; PY, patient‐years.

^a^ MACE cohort only.

There were 315 malignancies over 40251.60 PY (IR 0.78 per 100 PY) in the malignancy cohort (Table 2).

### 3.3 Variables Associated With MACE and Malignancies

Crude and adjusted HRs (95% CIs) for all independent variables that entered the final model are shown in Table S1.

**FIGURE 1:**
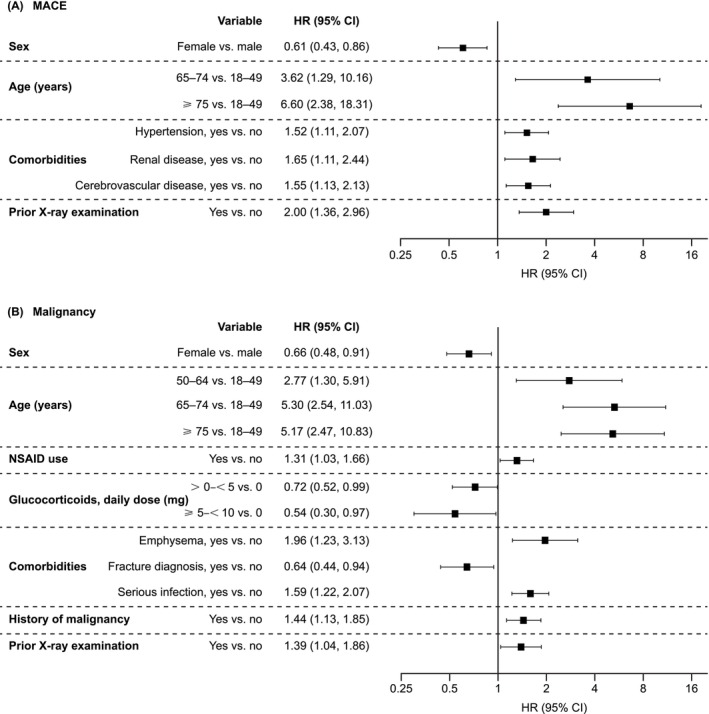
Statistically significant variables associated with (A) MACE and (B) malignancy. The primary analyses without laboratory tests included as risk factors are shown. Statistical significance was defined as *p* < 0.05. The covariate assessment window for sex and age was the index date; for NSAIDs and glucocorticoids, it was the period from 3 months prior to the index date; for comorbidities and prior X‐ray examination, it was the period from 1 year prior to the index date; and for history of malignancy, it was the period from the initial date in the database to 1 year prior to the index date. CI, confidence interval; HR, hazard ratio; MACE, major adverse cardiovascular events; NSAID, non‐steroidal anti‐inflammatory drug.

In the primary analysis, statistically significant (*p* < 0.05) variables associated with increased MACE risk were male sex, older age (≥ 65 years), hypertension, renal disease, cerebrovascular disease, and prior X‐ray examination (Figure 1A). Significant (*p* < 0.05) variables associated with increased malignancy risk were male sex, older age (≥ 50 years), NSAID use, emphysema, serious infection, history of malignancies, and prior X‐ray examination. Glucocorticoid use (< 10 mg/day) and fracture diagnosis were significantly associated with reduced malignancy risk (Figure 1B).

In the sensitivity analyses where laboratory tests were included as risk factors, significant (*p* < 0.05) variables associated with increased/reduced MACE and/or malignancy risk were similar to that of the primary analysis. Elevations in the tumor marker, squamous cell carcinoma antigen (SCCA), were associated with increased MACE risk, while elevations in neuron‐specific enolase (NSE) were associated with increased malignancy risk (Table S2). Similar risks of increased/reduced MACE and malignancy were observed in the sensitivity analyses using the missing‐indicator method, a wider definition of MACE, and the incident RA cohort (data not shown).

## 4 Discussion

Risk of safety outcomes such as MACE, malignancies, and herpes zoster have been reported to vary across geographic regions for RA medications, such as JAK inhibitors [18, 26]; therefore, it is important to evaluate underlying risk factors for safety outcomes at a country level. This observational cohort study set out to identify risk factors for MACE and malignancies (including NMSC) in patients with RA using real‐world data in Japan. The IR for MACE in the MACE cohort was 0.49 per 100 PY, while the IR for malignancy in the malignancy cohort was 0.78 per 100 PY. Significant variables associated with increased MACE risk were male sex, older age (≥ 65 years), hypertension, renal disease, cerebrovascular disease, and prior X‐ray examination; while male sex, older age (≥ 50 years), NSAID use, emphysema, serious infection, history of malignancies, and prior X‐ray examination were significantly associated with increased malignancy risk. In addition, glucocorticoid use (< 10 mg/day) and fracture diagnosis were significantly associated with reduced malignancy risk. The results were generally similar regardless of whether laboratory tests were included in the model, and in other sensitivity analyses (analyses using the missing‐indicator method, a wider definition of MACE, and the incident RA cohort). Interestingly, elevated SCCA and NSE were significantly associated with increased risk of MACE and malignancies, respectively, when laboratory tests were included in the model.

We observed a higher IR for malignancy (0.78 per 100 PY) versus MACE (0.49 per 100 PY) in the respective cohorts. This may be because 23.11% of patients in the malignancy cohort had a history of malignancy, in contrast to 0.42% of patients in the MACE cohort who had a history of MACE. In addition, 41.09%–41.31% of patients across cohorts were aged ≥ 75 years, which may have contributed to an increased susceptibility of events.

Male sex, older age, hypertension, renal disease, and NSAID use have previously been identified as risk factors for MACE and/or malignancies in studies of other patient populations with RA [6, 11, 13, 27, 28]. To our knowledge, cerebrovascular disease (MACE), prior X‐ray examination (MACE and malignancies), emphysema (malignancies), and serious infection (malignancies) have not previously been evaluated and/or identified as risk factors for these events in patients with RA [6, 11, 13, 27, 28]. As such, these variables warrant further investigation as potential specific risk factors in the Japanese RA population.

In this analysis, hypertension, renal disease, and cerebrovascular disease, but not cardiovascular disease, were associated with increased MACE risk. Specifically, cerebrovascular disease may be a proxy variable for history of MACE. However, this observation may be partially due to the low event rate for ischemic heart disease, for example, MI (35 of 214 MACE), and the high event rate for stroke (150 of 214 MACE) of all MACE that were observed in the study. A predominance of stroke versus ischemic heart disease may exist in the Japanese population, as a higher mortality rate for stroke compared with ischemic heart disease has been noted in patients with RA in Japan [29]. This contrasts with the United States, the United Kingdom, and France, which have shown a higher mortality rate for ischemic heart disease versus stroke in patients with RA [29].

Interestingly, prior X‐ray examination was identified as a variable associated with increased risk of both MACE and malignancies. This variable has not been identified as a risk factor for these adverse events in previous studies of patients with RA [6, 11, 13, 27, 28]. These associations require cautious interpretation; however, rather than being a risk factor as such, patients experiencing MACE and/or malignancies likely had a higher chance of undergoing X‐ray examination because of additional symptoms such as chest pain and weight loss. Across MACE/malignancy cohorts in this study, most (67.76%–69.32%) patients with RA had undergone X‐ray examination 1 year before the index date, although the anatomical locations of these examinations were not captured in this analysis. Further investigation is required to understand the mechanisms underpinning these associations and to detect confounding factors related to symptom‐driven diagnostic investigations such as frequency of X‐rays, total number of X‐rays, time interval between X‐rays, and MACE and malignancy diagnoses. Additionally, while an increased risk of malignancy has been postulated as a consequence of the exposure to medical imaging, namely prior computed tomography (CT) examination [30], prior CT examination was not identified as a risk factor in this analysis. In the sensitivity analyses with laboratory tests included in the model, elevated SCCA was associated with increased risk of MACE. Although higher SCCA is a marker for squamous cell carcinoma itself, a greater risk of developing NMSC has been reported in a study in the Taiwan general population requiring hemodialysis [31]. Therefore, this association may have been observed because SCCA elevations can be recorded in patients with renal failure. Indeed, renal disease was also associated with MACE in this analysis.

With regard to the risk of malignancy, although emphysema has not been reported as a risk factor in previous studies [6, 28], this may be linked to associations that have previously been identified with smoking [32]. The high level of missing data with regard to smoking in this analysis likely reduced any potential associations with this variable and was a limiting factor of this study. Surprisingly, serious infection was also associated with a significantly increased risk of malignancy, which may arise in those who are immunocompromised. In addition, glucocorticoid use (< 10 mg/day) and fractures were associated with reduced malignancy risk. The impact of glucocorticoids on cancer is thought to be largely dependent on cancer type, although glucocorticoids are commonly used in the treatment of lymphoma by inducing cell apoptosis [33]. Nevertheless, further studies are required to elucidate the potential mechanisms underpinning these associations in patients with RA in Japan.

This study should be discussed in the context of some limitations. This was an observational study, without a comparator arm, which included unmeasured confounding and misclassification bias for outcomes, exposures, and covariates. The high level of missing data for body mass index and smoking (> 70% across cohorts) may have reduced the potential associations with these variables, although it should be noted that these variables were imputed for missing data by the multiple imputation method. In order to assess glucocorticoid use as a risk factor, patients with RA in this analysis included those who were prescribed ≥ 1 DMARD or glucocorticoid. This was with the knowledge that RA diagnosis + DMARD and/or glucocorticoid, versus RA diagnosis + any DMARD, using a physician diagnosis as the gold standard, could maintain an acceptable, albeit lower, positive predictive value (PPV), as previously reported by an analysis of a Japanese hospital‐based validation study (PPV [95% CI] 77.6 [73.5, 81.8] vs. 88.3 [84.6, 91.2]) [23]. If a patient attended a different hospital or clinic after discharge, follow‐up on the patient's record was not possible. As an electronic medical records‐based nationwide database was used as the data source in this study, the generalizability of the results may be reduced. Lastly, the absence of prior X‐rays is outside standard practice; therefore, a comparison between the presence and absence of prior X‐rays may be required to detect confounding factors.

## 5 Conclusion

To the best of our knowledge, this is one of the first studies to comprehensively identify risk factors associated with MACE and malignancy in patients with RA in real‐world data in Japan. Male sex, older age (≥ 65 years), hypertension, renal disease, cerebrovascular disease, and prior X‐ray examination were associated with increased MACE risk, while male sex, older age (≥ 50 years), NSAID use, emphysema, serious infection, history of malignancies, and prior X‐ray examination were associated with increased malignancy risk. Further studies are required to evaluate the underlying mechanisms of some associations, such as that of prior X‐ray examination, that were identified in this analysis in patients with RA from real‐world data in Japan.

## Author Contributions

Study conceptualization/design and data analysis: Kunihiro Yamaoka, Naonobu Sugiyama, Masato Hoshi, Joo‐Young Jo, and Toshitaka Hirano Data acquisition: Naonobu Sugiyama, Masato Hoshi, Joo‐Young Jo, and Toshitaka Hirano. All authors (Kunihiro Yamaoka, Naonobu Sugiyama, Masato Hoshi, Joo‐Young Jo, Kichul Shin, and Toshitaka Hirano) contributed to the interpretation of the data, were involved in drafting the article or reviewing it critically for important intellectual content, and approved the final version to be submitted for publication.

## Conflicts of Interest

K.Y. has received grants and/or research support, and speakers' fees and/or honoraria, from AbbVie, Actelion Japan, Asahi Kasei, Astellas, Ayumi, Boehringer Ingelheim Japan, Bristol Myers Squibb, Chugai, Daiichi Sankyo, Eisai, Eli Lilly Japan, Gilead Sciences, GSK, Hisamitsu, Janssen, Japan Tobacco Inc., Mitsubishi Tanabe Pharma, MSD, Nippon Kayaku, Nippon Shinyaku, Ono, Otsuka, Pfizer Inc., Sanofi, Takeda, and Teijin. N.S., M.H, and T.H. are employees and stockholders of Pfizer Japan Inc. J.‐Y.J. is an employee and stockholder of Pfizer Pharmaceuticals Korea Ltd. K.S. has received research support, and speakers' fees and/or honoraria, from AbbVie, Bristol Myers Squibb, Celltrion, Eisai, Eli Lilly Korea, Janssen, Novartis, Pfizer Inc., Samsung Bioepis, and Yuhan.

## Supporting information


Data S1.

## Data Availability

Upon request, and subject to review, Pfizer will provide the data that support the findings of this study. Subject to certain criteria, conditions, and exceptions, Pfizer may also provide access to the related individual de‐identified participant data. See https://www.pfizer.com/science/clinical‐trials/trial‐data‐and‐results for more information.
