# Supplementary material for: Risk Factors Associated With Major Adverse Cardiovascular Events and Malignancies in Patients With Rheumatoid Arthritis in a Real‐World Setting in Japan
Source: Int J Rheum Dis. 2024 Dec 9;27(12):e15448. doi: 10.1111/1756-185X.15448 (PMC11626545; doi:10.1111/1756-185X.15448)
Supplement: Supplementary file 1 — Data S1. [file APL-27-e15448-s001.pdf]

# SUPPLEMENTARY MATERIAL

## Supplementary Figure

FIGURE S1 Patient flow for the MACE and malignancy cohorts.

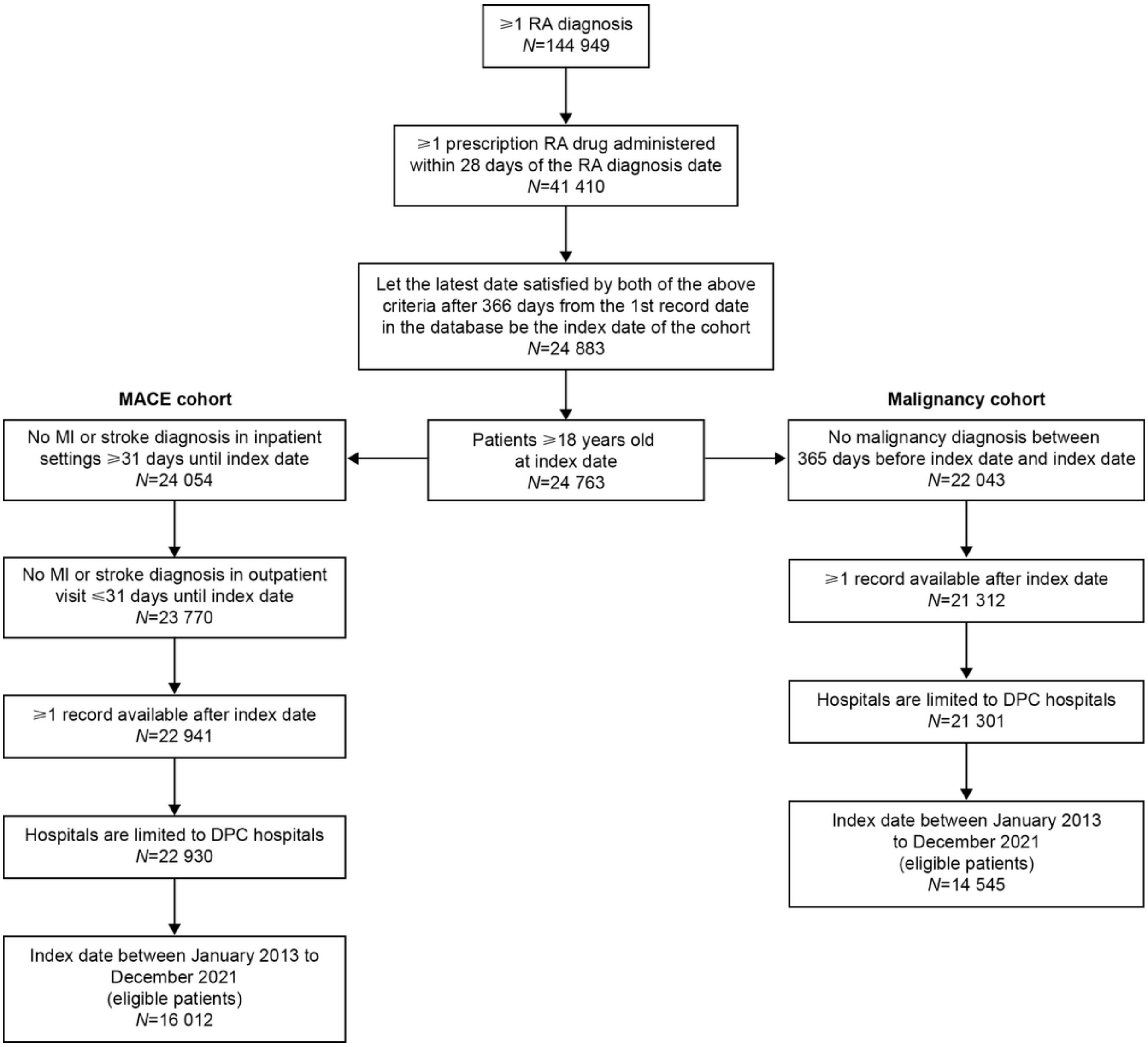


DPC, diagnostic procedure combination; MACE, major adverse cardiovascular events; MI, myocardial infarction; *N*, number of eligible patients within the criteria; RA, rheumatoid arthritis.

## Supplementary Tables

TABLE S1 Variables that entered the final Cox regression model with crude and adjusted HRs.

| | MACE cohort (*N*=16 012) | | Malignancy cohort (*N*=14 545) | |
|---|---|---|---|---|
| | **Crude HR (95% CI)** | **Adjusted HR (95% CI)** | **Crude HR (95% CI)** | **Adjusted HR (95% CI)** |
| Sex (female vs. male) | 0.59 (0.45, 0.77) | 0.62 (0.44, 0.87) | 0.51 (0.41, 0.64) | 0.65 (0.48, 0.89) |
| Age, years (vs. 18–49) | | | | |
| 50–64 | 2.60 (0.89, 7.59) | 2.27 (0.77, 6.69) | 3.03 (1.43, 6.41) | 2.72 (1.28, 5.80) |
| 65–74 | 5.13 (1.85, 14.21) | 3.55 (1.26, 9.98) | 6.21 (3.03, 12.72) | 5.21 (2.51, 10.82) |
| ≥75 | 10.90 (4.01, 29.67) | 6.55 (2.36, 18.21) | 5.50 (2.69, 11.28) | 5.04 (2.41, 10.55) |
| BMI, kg/m$^2$ (vs. <18.5) | | | | |
| ≥18.5–<25 | 0.94 (0.54, 1.63) | 1.03 (0.59, 1.81) | 1.00 (0.62, 1.61) | 1.04 (0.64, 1.69) |
| ≥25 | 0.77 (0.37, 1.60) | 0.95 (0.45, 2.04) | 1.00 (0.50, 1.99) | 1.12 (0.54, 2.31) |
| Smoking index,[a] (vs. non-smoker) | | | | |
| 1–399 | 1.13 (0.54, 2.35) | 1.07 (0.50, 2.30) | 1.12 (0.59, 2.14) | 0.91 (0.46, 1.80) |
| 400–799 | 1.55 (0.95, 2.54) | 1.28 (0.74, 2.22) | 1.63 (1.02, 2.61) | 1.17 (0.68, 1.99) |
| ≥800 | 1.25 (0.63, 2.49) | 0.77 (0.34, 1.72) | 2.01 (1.26, 3.20) | 1.06 (0.58, 1.94) |
| Comorbidities (yes vs. no) | | | | |
| Cerebrovascular disease | 2.34 (1.74, 3.14) | 1.58 (1.15, 2.16) | 1.25 (0.95, 1.64) | 1.09 (0.81, 1.45) |
| Diabetes | 1.58 (1.15, 2.17) | 1.07 (0.76, 1.50) | 1.40 (1.07, 1.84) | 1.15 (0.86, 1.54) |
| Emphysema | 1.64 (0.87, 3.11) | 0.91 (0.46, 1.81) | 3.45 (2.27, 5.23) | 1.97 (1.23, 3.14) |
| Fracture diagnosis | 1.73 (1.24, 2.41) | 1.40 (0.99, 1.98) | 0.72 (0.50, 1.04) | 0.64 (0.44, 0.94) |
| Hypertension | 2.32 (1.76, 3.05) | 1.51 (1.11, 2.06) | 0.99 (0.79, 1.25) | 0.77 (0.59, 1.00) |
| Hyperlipidemia | 1.44 (1.09, 1.90) | 0.96 (0.71, 1.31) | 1.04 (0.82, 1.33) | 0.98 (0.75, 1.28) |

| | | | | |
|---|---|---|---|---|
| Interstitial lung disease | – | – | 1.62 (1.22, 2.17) | 1.35 (0.96, 1.89) |
| Ischemic heart disease | 1.44 (1.03, 1.99) | 0.81 (0.57, 1.15) | 1.25 (0.95, 1.66) | 0.98 (0.73, 1.34) |
| Renal disease | 2.33 (1.61, 3.37) | 1.67 (1.12, 2.47) | 0.79 (0.49, 1.27) | 0.71 (0.43, 1.17) |
| Unspecified chronic bronchitis | – | – | 0.97 (0.63, 1.50) | 0.74 (0.47, 1.17) |
| Nontuberculous mycobacteriosis | 3.37 (0.83, 13.68) | 2.01 (0.47, 8.67) | – | – |
| Other COPD | 1.76 (0.96, 3.25) | 1.27 (0.66, 2.44) | 2.60 (1.67, 4.06) | 1.36 (0.83, 2.24) |
| Pulmonary embolism | – | – | 3.46 (0.86, 13.99) | 1.98 (0.47, 8.38) |
| Serious infection | 1.24 (0.90, 1.71) | 0.76 (0.54, 1.07) | 1.80 (1.41, 2.29) | 1.52 (1.16, 1.99) |
| Systemic lupus erythematosus | – | – | 0.56 (0.26, 1.18) | 0.89 (0.41, 1.92) |
| Systemic sclerosis | – | – | 0.36 (0.09, 1.45) | 0.46 (0.11, 1.87) |
| Medications,[b] (yes vs. no or >0 mg vs. 0 mg) | | | | |
| NSAIDs | 0.87 (0.67, 1.15) | 0.81 (0.61, 1.08) | 1.30 (1.03, 1.64) | 1.33 (1.05, 1.69) |
| Glucocorticoids, daily dose, mg | | | | |
| >0–<5 | 1.56 (1.16, 2.10) | 1.45 (0.99, 2.12) | 0.82 (0.65, 1.03) | 0.72 (0.52, 0.99) |
| ≥5–<10 | 1.58 (0.95, 2.63) | 1.53 (0.88, 2.66) | 0.57 (0.33, 0.99) | 0.54 (0.30, 0.98) |
| ≥10 | 0.91 (0.42, 1.99) | 0.81 (0.35, 1.85) | 0.94 (0.55, 1.61) | 0.79 (0.44, 1.43) |
| bDMARDs | 0.86 (0.47, 1.58) | 1.15 (0.61, 2.15) | 0.59 (0.33, 1.05) | 0.61 (0.34, 1.11) |
| csDMARDs | 0.76 (0.58, 1.00) | 1.34 (0.90, 1.99) | 1.17 (0.93, 1.47) | 1.07 (0.74, 1.56) |
| Methotrexate, weekly dose, mg | | | | |
| >0–≤8 | 0.62 (0.44, 0.87) | 0.76 (0.51, 1.14) | 1.02 (0.79, 1.30) | 0.97 (0.71, 1.32) |
| >8 | 0.29 (0.09, 0.92) | 0.37 (0.11, 1.20) | 1.41 (0.87, 2.28) | 1.56 (0.92, 2.63) |
| JAK inhibitor | – | – | 1.97 (0.27, 14.16) | 1.38 (0.19, 10.17) |

| | | | | |
|---|---|---|---|---|
| Calcineurin inhibitor | 0.95 (0.55, 1.63) | 0.82 (0.46, 1.46) | 0.91 (0.58, 1.43) | 0.82 (0.50, 1.33) |
| History of MACE (yes vs. no) | 6.87 (1.68, 28.03) | 3.08 (0.72, 13.11) | – | – |
| History of malignancy (yes vs. no) | – | – | 1.61 (1.27, 2.04) | 1.45 (1.13, 1.86) |
| Prior imaging examination (yes vs. no) | | | | |
| CT | 2.29 (1.74, 3.00) | 1.29 (0.93, 1.79) | 1.62 (1.29, 2.03) | 1.14 (0.86, 1.51) |
| X-ray | 2.41 (1.72, 3.37) | 2.02 (1.37, 2.99) | 1.58 (1.23, 2.02) | 1.42 (1.06, 1.90) |
| MRI | 1.46 (1.05, 2.02) | 0.89 (0.63, 1.26) | – | – |
| Cholesterol (LDL-c/HDL-c) (increased vs. normal range) | 1.34 (0.83, 2.15) | 1.06 (0.65, 1.73) | 1.15 (0.76, 1.73) | 0.98 (0.65, 1.49) |
| Laboratory tests related to MACE (increased vs. normal range) | | | | |
| CK | – | – | 2.11 (1.21, 3.68) | 1.78 (0.98, 3.24) |
| LDH | 1.46 (1.03, 2.08) | 1.18 (0.81, 1.71) | 1.33 (0.99, 1.78) | 1.08 (0.78, 1.51) |
| WBC | – | – | 1.26 (0.94, 1.69) | 1.07 (0.78, 1.46) |
| Laboratory tests related to malignancy (increased vs. normal range) | | | | |
| CA19-9 | 2.34 (0.74, 7.37) | 1.57 (0.44, 5.60) | 2.93 (1.09, 7.90) | 1.30 (0.43, 3.93) |
| CEA | 2.17 (1.06, 4.42) | 1.14 (0.50, 2.62) | 2.61 (1.38, 4.91) | 1.49 (0.70, 3.16) |
| CYFRA | – | – | 4.51 (1.44, 14.16) | 1.74 (0.51, 5.95) |
| NSE | 9.85 (1.36, 71.24) | 6.05 (0.73, 50.23) | 12.67 (1.76, 91.07) | 12.28 (1.57, 95.82) |
| SCCA | 11.11 (2.72, 45.33) | 6.17 (1.35, 28.10) | – | – |
| sIL-2R | 2.05 (1.17, 3.61) | 1.30 (0.72, 2.36) | – | – |

| | | | | |
|---|---|---|---|---|
| Laboratory tests related to interstitial lung disease (increased vs. normal range) | | | | |
| KL-6 | – | – | 1.55 (0.99, 2.41) | 0.82 (0.47, 1.44) |
| SPD | 2.19 (1.12, 4.29) | 1.48 (0.72, 3.04) | 2.21 (1.27, 3.86) | 1.37 (0.70, 2.68) |

*Note:* Variables that entered the final model with laboratory tests included as risk factors are shown. Crude HRs were from separate univariate models and adjusted HRs were from the final models after the systematic variable selection procedure.

Abbreviations: bDMARD, biologic disease-modifying antirheumatic drug; BMI, body mass index; CA19-9, carbohydrate antigen 19-9; CEA, carcinoembryonic antigen; CI, confidence interval; CK, creatine kinase; COPD, chronic obstructive pulmonary disease; csDMARD, conventional synthetic disease-modifying antirheumatic drug; CT, computed tomography; CYFRA, cytokeratin-19 fragments; HDL-c, high-density lipoprotein-cholesterol; HR, hazard ratio; JAK, Janus kinase; KL-6, Krebs von den Lungen-6; LDH, lactate dehydrogenase; LDL-c, low-density lipoprotein-cholesterol; MACE, major adverse cardiovascular events; MRI, magnetic resonance imaging; *N*, total number of eligible patients; NSAID, non-steroidal anti-inflammatory drug; NSE, neuron-specific enolase; SCCA, squamous-cell carcinoma antigen; sIL-2R, soluble interleukin-2 receptor; SPD, surfactant protein D; WBC, white blood cell.

[a]Smoking index was calculated by the formula: the number of cigarettes smoked per day × years of smoking.

[b]Within 3 months prior to the index date.

TABLE S2 Statistically significant variables associated with MACE and/or malignancy in the sensitivity analysis with laboratory tests included in the final Cox regression model.

| | **MACE cohort (*N*=16 012)** | **Malignancy cohort (*N*=14 545)** |
|---|---|---|
| | **HR (95% CI)** | **HR (95% CI)** |
| Sex (female vs. male) | 0.62 (0.44, 0.87) | 0.65 (0.48, 0.89) |
| Age, years (vs. 18–49) | | |
| 50–64 | – | 2.72 (1.28, 5.80) |
| 65–74 | 3.55 (1.26, 9.98) | 5.21 (2.51, 10.82) |
| ≥75 | 6.55 (2.36, 18.21) | 5.04 (2.41, 10.55) |
| Comorbidities (yes vs. no) | | |
| Cerebrovascular disease | 1.58 (1.15, 2.16) | – |
| Fracture diagnosis | – | 0.64 (0.44, 0.94) |
| Hypertension | 1.51 (1.11, 2.06) | – |
| Renal disease | 1.67 (1.12, 2.47) | – |
| Serious infection | – | 1.52 (1.16, 1.99) |
| Medications (yes vs. no or >0 mg vs. 0 mg) | | |
| NSAIDs | – | 1.33 (1.05, 1.69) |
| Glucocorticoids, daily dose, mg | – | – |
| >0–<5 | – | 0.72 (0.52, 0.99) |
| ≥5–<10 | – | 0.54 (0.30, 0.98) |
| ≥10 | – | 0.79 (0.44, 1.43) |
| History of malignancy (yes vs. no) | – | 1.45 (1.13, 1.86) |
| Prior X-ray examination (yes vs. no) | 2.02 (1.37, 2.99) | 1.42 (1.06, 1.90) |

| | | |
|---|---|---|
| Laboratory tests related to malignancy | | |
| (increased vs. normal range) | | |
| SCCA | 6.17 (1.35, 28.10) | – |
| NSE | – | 12.28 (1.57, 95.82) |

Abbreviations: CI, confidence interval; HR, hazard ratio; MACE, major adverse cardiovascular events; *N*, total number of eligible patients; NSAID, non-steroidal anti-inflammatory drug; NSE, neuron-specific enolase; SCCA, squamous cell carcinoma antigen.

Statistical significance was defined as $p<0.05$.

Missing values for laboratory test results were treated as ‘normal’.

The covariate assessment window for sex and age was the index date, for comorbidities and X-rays, it was the period from 1 year prior to the index date to the index; for NSAIDs, glucocorticoids, NSE, and SCCA, it was the period from 3 months prior to the index date to the index date; and for past history of malignancies, it was the period from the initial date in the database to 1 year prior to the index date.
